# Supplementary material for: Drivers of long-lasting insecticide-treated net utilisation and parasitaemia among under-five children in 13 States with high malaria burden in Nigeria
Source: PLoS One. 2022 May 6;17(5):e0268185. doi: 10.1371/journal.pone.0268185 (PMC9075637; doi:10.1371/journal.pone.0268185)
Supplement: Attachment — Submitted filename: PLOS ONE - Reviewers comment from Dr. C. M. Egbuche.pdf [file pone.0268185.s001.pdf]

PLOS ONE

Drivers of long-lasting insecticide-treated net utilisation and parasitaemia among under-five children in 13 States with high malaria burden in Nigeria.

Manuscript Number: PONE-D-21-27138

ABSTRACT –

Methods

Line 28: Data from…………….were obtained and analyzed.

You didn't state the role of the 2015 data set (to categorize malaria burdens in states, to compare with 2018 data for increase or reduced parasitaemia?)

Line 29 - 30: The thirteen states studied were stratified into two based on increased or reduced parasitaemia between 2015 and 2018. (Remove whether they had…).

Line 43: Last paragraph of the result….. 2.70) were more likely to have parasitaemia. NOT were more likely of parasitaemia.

INTRODUCTION

Line 70: economic loses: out of pocket payment, …….. (Add colon before listing).

Line 93: For instance, parasitaemia was reported to be more prevalent………

Line 95: ……..those from rich households were reported to have lower risks (5)

Line 95 – 96: remove the statement 'this is a direct reflection of the fact that malaria is a poverty related disease'.

Line 103 – 105: Four of these states had increased parasitaemia between 2015 and 2018, while the remaining nine experienced a decrease (Citation is required).

Line 106: What is programmatic intervention?

Line 109 – 110: the statement there is not necessary.

METHODS

Line 113: This study involves analysis of secondary data obtained from the …..OR This is a retrospective study that utilized two national data sets. The 2015 data set on NMIS was used to categorize states as having high or low malaria burden, and also to compare with 2018 data for indicating increased or reduced paraasitaemia……..

Line 113 – 115: collapse into the subtitle 'description of data sources'

Line 119: 49 years old

Line 122: specify the sample size ; not over 8,000 households

Line 126: 15 – 45 years

Line 126 – 130: no indication of study duration, from which month to ?

Line 132: The data sets from 2015 NMIS and 2018 NDHS were both obtained from stratified samples selected in two stages.

Line 134: write EAs in full

Line 144: men aged 15 to 59 years were not mentioned before ???????

Line 144: biomarker sample? Do you mean blood sample?

Line 148 – 152: did you select the states based on rural / urban stratification? and/or on high malaria burden? Which of the data sets provided guide for the selection or description of the study population?

Line 160: Write GF in full
Line 173: Wealth quintiles? Do you mean quartiles?

RESULTS

Line 209 – 214: you did not clearly explain Table 2.
Line 211: ………., while close to one quarter. Please specify the value.
Line 216: first column in Table 2 has no title. I suggest 'Variables / categories'
Line 216: titles of the 2nd and 3rd panels in Table 2 are not clear. 'state with increase'? 'states with reduction'?

Line 246 – 252: where is the result described? There is no reference to any Table or Figure.

Line 271: …….24.2 % respectively in 2018. Remove comma.
Line 272: is the difference in prevalence between 2015 and 2018 data the same as prevalence ratio?
Line 275: where is the Figure 3?

Line 281 – 282: where is the reference group in that statement? 'In contrast, stunted (OR=1.16, 95%CI: 1.00-1.34) and wasted (OR=1.40, 95% CI:1.05-1.88) children were more likely of sleeping under LLIN, than ………..

Your result presentation focused only on 2018 data. No Table presented comparative prevalence in 2015 and 2018 to show increase or decrease in prevalence.

DISCUSSION

Line 390 – 391: ……..global scales (Allwell-Brown, 2017). This citation format is not consistent with the one you have been using. Check other parts of this work and make the citation uniform.

Line 407: an increase in parasitaemia level over. What is OVER in the sentence?
Line 428 – 429: Recast this phrase 'Malaria eliminating countries'

Line 438 – 439: check the punctuations in that statement.

REVIEWER: Dr C. M. Egbuche
