# Supplementary material for: Drivers of long-lasting insecticide-treated net utilisation and parasitaemia among under-five children in 13 States with high malaria burden in Nigeria
Source: PLoS One. 2022 May 6;17(5):e0268185. doi: 10.1371/journal.pone.0268185 (PMC9075637; doi:10.1371/journal.pone.0268185)
Supplement: Attachment — Submitted filename: PLOS ONE ReviewersComments AND RESPONSES.docx [file pone.0268185.s002.docx]

Reviewer #1: ABSTRACT –
Methods
Line 28: Data from…………….were obtained and analyzed.
You didn’t state the role of the 2015 data set (to categorize malaria burdens in states, to compare with 2018 data for increase or reduced parasitaemia?)

RESPONSE: Thank you. We have stated the role of 2015 data.
Line 29 - 30: The thirteen states studied were stratified into two based on increased or reduced parasitaemia between 2015 and 2018. (Remove whether they had…).

RESPONSE: This has been done. Thank you
Line 43: Last paragraph of the result….. 2.70) were more likely to have parasitaemia. NOT were more likely of parasitaemia.
RESPONSE: Thank you, correction has been done as recommended
INTRODUCTION

Line 70: economic loses: out of pocket payment, …….. (Add colon before listing).

RESPONSE: thank you, colon has been added before listing
Line 93: For instance, parasitaemia was reported to be more prevalent………

RESPONSE: Thank, edits has been adopted
Line 95: ……..those from rich households were reported to have lower risks (5)

RESPONSE: Thank you, suggestion has been adopted
Line 95 – 96: remove the statement ‘this is a direct reflection of the fact that malaria is a poverty related disease’.

RESPONSE: Statement has been removed
Line 103 – 105: Four of these states had increased parasitaemia between 2015 and 2018, while the remaining nine experienced a decrease (Citation is required).
RESPONSE: Citation has been provided

Line 106: What is programmatic intervention?

RESPONSE: We have revised it for better clarity
Line 109 – 110: the statement there is not necessary.
RESPONSE: This statement was meant to clearly described the objectives of the paper. We retained it so that readers can easily link our results with the objectives.
METHODS

Line 113: This study involves analysis of secondary data obtained from the …..OR This is a retrospective study that utilized two national data sets. The 2015 data set on NMIS was used to categorize states as having high or low malaria burden, and also to compare with 2018 data for indicating increased or reduced paraasitaemia……..

RESPONSE: Thank you for these suggested revisions. We have adopted it and edited the section accordingly.

Line 113 – 115: collapse into the subtitle ‘description of data sources’
RESPONSE: This has been done
Line 119: 49 years old

RESPONSE: This correction has been made. Thank you
Line 122: specify the sample size ; not over 8,000 households

RESPONSE: the actual no of households have been stated
Line 126: 15 – 45 years

RESPONSE: Thank you for pointing out this typo. We have corrected it
Line 126 – 130: no indication of study duration, from which month to ?

RESPONSE: This has been provided
Line 132: The data sets from 2015 NMIS and 2018 NDHS were both obtained from stratified samples selected in two stages.

RESPONSE: thank you for the suggested revision which we have adopted
Line 134: write EAs in full

RESPONSE: EA has been written in full
Line 144: men aged 15 to 59 years were not mentioned before ???????

RESPONSE: Usually, DHS surveys include men. However, we have deleted the mention of men in this manuscript because we did not analyse men’s data.
Line 144: biomarker sample? Do you mean blood sample?

RESPONSE: Yes, we meant blood sample. We have replaced biomarker with “blood”
Line 148 – 152: did you select the states based on rural / urban stratification? and/or on high malaria burden? Which of the data sets provided guide for the selection or description of the study population?
RESPONSE: States were selected based on malaria burden. Both datasets provided guide for description of study population
Line 160: Write GF in full

RESPONSE: Apology for the mistake. GF has been deleted

Line 173: Wealth quintiles? Do you mean quartiles?

RESPONSE: This is wealth quintiles because it has 5 categories. Quartiles would have only 4.

RESULTS

Line 209 – 214: you did not clearly explain Table 2.

RESPONSE: Pls, note that Table 2 was further described under “utilisation of LLIN” and “prevalence of parasitaemia”

Line 211: ………., while close to one quarter. Please specify the value.

RESPONSE: The actual value has been stated
Line 216: first column in Table 2 has no title. I suggest ‘Variables / categories’

RESPONSE: Thank you for the suggestion. We have revised the column title as recommended
Line 216: titles of the 2nd and 3rd panels in Table 2 are not clear. ‘state with increase’? ‘states with reduction’?
RESPONSE: The column titles has been made clearer
Line 246 – 252: where is the result described? There is no reference to any Table or Figure.
RESPONSE: We have provided reference to Tables 4a, 4b and Figure 3
Line 271: …….24.2 % respectively in 2018. Remove comma.

RESPONSE: Thank you, Comma has been removed
Line 272: is the difference in prevalence between 2015 and 2018 data the same as prevalence ratio?

RESPONSE: No, they are not the same
Line 275: where is the Figure 3?
RESPONSE: Figures are provided as separate files but included in the manuscript for review
Line 281 – 282: where is the reference group in that statement? ‘In contrast, stunted (OR=1.16, 95%CI: 1.00-1.34) and wasted (OR=1.40, 95% CI:1.05-1.88) children were more likely of sleeping under LLIN, than ………..
RESPONSE: Sentence has been revised. Thank you
Your result presentation focused only on 2018 data. No Table presented comparative prevalence in 2015 and 2018 to show increase or decrease in prevalence.

RESPONSE: Figure 3 utilized data from both MIS 2015 and DHS 2018. Besides, we have added tables 4a and 4b which showed comparison of LLIN utilization and parasitaemia prevalence as well as percentage changes in the 13 study States between 2015 and 2018

DISCUSSION

Line 390 – 391: ……..global scales (Allwell-Brown, 2017). This citation format is not consistent with the one you have been using. Check other parts of this work and make the citation uniform.
RESPONSE: Reference style has been corrected for consistency
Line 407: an increase in parasitaemia level over. What is OVER in the sentence?

RESPONSE: We have deleted the extraneous word “Over”
Line 428 – 429: Recast this phrase ‘Malaria eliminating countries’
RESPONSE: The phrase has been recasted
Line 438 – 439: check the punctuations in that statement.

RESPONSE: Thank you the error in punctuation have been corrected
