# Supplementary material for: Drivers of long-lasting insecticide-treated net utilisation and parasitaemia among under-five children in 13 States with high malaria burden in Nigeria
Source: PLoS One. 2022 May 6;17(5):e0268185. doi: 10.1371/journal.pone.0268185 (PMC9075637; doi:10.1371/journal.pone.0268185)
Supplement: Attachment — Submitted filename: PONE-D-21-27138R1.docx [file pone.0268185.s003.docx]

COMMENTS TO AUTHOR(S)

The manuscript is sound but there is need for minor revision in some components as elaborated in the comments by the reviewers.

REVIEWER 1:

COMMENTS

The author has effected all the corrections pointed out.

Aside that, I noted the following minor corrections which I did not point out the first time (Please I apologize for that).

1. Add title to the first column in table 3. I suggest ‘Variables / categories’
2. Redo the first and second rows of Table 5 as shown below.
3. Redo the first and second rows of Table 6 as shown below

The author can actually lift the Tables (Tables 5 and 6) as presented here. I have only adjusted the rows for the author. I did not touch the data.

The manuscript is recommended for publication.

Thanks.

**Dr. Chukwudi Michael Egbuche**

Table 5

| **Individual child/maternal characteristics** | **Unadjusted OR (95% CI)** | **p value** | **All States** | | **States with Malaria Increase** | | **States with Malaria reduction** | |
| --- | --- | --- | --- | --- | --- | --- | --- | --- |
|  |  |  | **Adjusted OR (95% CI)** | **p value** | **Adjusted OR (95% CI)** | **p value** | **Adjusted OR (95% CI)** | **p value** |
| *Child's age (Months)* |  |  |  |  |  |  |  |  |
| 0 - 11 | 1.00 |  |  |  |  |  |  |  |
| 12 - 23 | 0.99 (0.80 - 1.23) | 0.96 |  |  |  |  |  |  |
| 24 - 59 | 0.85 (0.72 - 1.02) | 0.078 |  |  |  |  |  |  |
| *Child's sex* |  |  |  |  |  |  |  |  |
| Male | 1.00 |  |  |  |  |  |  |  |
| Female | 0.97 (0.85 - 1.12) | 0.689 |  |  |  |  |  |  |
| *Relationship to head of household* |  |  |  |  |  |  |  |  |
| Child | 1.00 |  |  |  |  |  |  |  |
| Grandchild | 0.56 (0.42 - 0.75)* | <0.001 | 5.35 (1.09-26.19)* | 0.039 | 4.14 (0.47 - 36.62) | 0.202 | 4.20 (0.24-72.74) | 0.324 |
| Other relative | 0.56 (0.38 - 0.82)* | 0.003 | 0.33 (0.11 - 0.94)* | 0.038 | 0.22 (0.07 - 0.76)* | 0.016 | 5.22 (0.25-107.61) | 0.284 |
| *Fever in past 2 weeks (Yes vs No)* | 1.09 (0.92 - 1.28) | 0.328 |  |  |  |  |  |  |
| *Stunting (Yes vs No)* | 1.16 (1.00 - 1.34) | 0.055 | 0.75 (0.50-1.13) | 0.166 | 0.82 (0.51-1.31) | 0.406 | 0.64 (0.22 - 1.83) | 0.408 |
| *Wasting (Yes vs No)* | 1.40 (1.05 - 1.88)* | 0.022 | 1.25 (0.57-2.72) | 0.582 | 0.93 (0.38-2.27) | 0.881 | 4.18 (0.34 - 51.65) | 0.264 |
| *Age of mother (Years)* |  |  |  |  |  |  |  |  |
| < 25 | 1.00 |  |  |  |  |  |  |  |
| 25-34 | 0.77 (0.64 - 0.93)* | 0.006 | 0.92 (0.53-1.59) | 0.753 | 0.78 (0.42 - 1.45) | 0.429 | 3.60 (0.98 - 13.27) | 0.054 |
| 35-49 | 0.68 (0.55 - 0.84)* | <0.001 | 0.70 (0.38-1.30) | 0.259 | 0.65 (0.33-1.29) | 0.218 | 3.39 (0.81-14.11) | 0.094 |
| *Mother's education* |  |  |  |  |  |  |  |  |
| None | 1.00 |  |  |  |  |  |  |  |
| Primary | 1.19 (0.95 - 1.50) | 0.138 |  |  |  |  |  |  |
| Secondary/higher | 0.97 (0.78 - 1.21) | 0.811 |  |  |  |  |  |  |
| *Mother's occupation* |  |  |  |  |  |  |  |  |
| Not working | 1.00 |  |  |  |  |  |  |  |
| White collar job | 1.09 (0.81 - 1.47) | 0.559 |  |  |  |  |  |  |
| Sales | 0.86 (0.73 - 1.02) | 0.09 |  |  |  |  |  |  |
| Agric/manual work | 0.84 (0.67 - 1.06) | 0.144 |  |  |  |  |  |  |
| *Exposure to radio/television* |  |  |  |  |  |  |  |  |
| Not at all | 1.00 |  |  |  |  |  |  |  |
| less than once a week | 0.75 (0.61 - 0.93)* | 0.007 | 0.46 (0.26-0.81)** | 0.007 | 0.65 (0.32-1.28) | 0.212 | 0.13 (0.03 - 0.54)* | 0.005 |
| at least once a week | 0.79 (0.65 - 0.96)* | 0.02 | 0.61 (0.34-1.10) | 0.098 | 0.84 (0.43-1.67) | 0.621 | 0.58 (0.13 - 2.57) | 0.475 |
| *Health Insurance* |  |  |  |  |  |  |  |  |
| Yes vs No | 1.17 (0.65 - 2.10) | 0.599 |  |  |  |  |  |  |
| **Perceptions/opinions about malaria** |  |  |  |  |  |  |  |  |
| Preventative medicine keeps baby healthy | 1.31 (1.06 - 1.63)* | 0.014 | 1.71 (0.60-4.90) | 0.318 |  |  | 19.92 (4.15-95.67)* | <0.001 |
| Malaria can be fully cured by medicine | 1.17 (0.97 - 1.41) | 0.095 |  |  |  |  |  |  |
| Malaria can lead to death | 1.31 (1.12 - 1.52)* | 0.001 | 1.55 (0.95-2.63) | 0.089 | 2.52 (1.32 - 4.83)* | 0.005 | 0.63 (0.19 - 2.06) | 0.448 |
| No worry about malaria due to easy treatment | 1.01 (0.86 - 1.18) | 0.906 |  |  |  |  |  |  |
| I know people sick with malaria | 1.08 (0.93 - 1.26) | 0.307 |  |  |  |  |  |  |
| Only weak children can die from malaria | 1.67 (1.41 - 1.98)* | <0.001 | 1.83 (1.10-3.10)* | 0.023 | 0.99 (0.54-1.83) | 0.9995 | 15.64 (2.88-85.05)* | 0.001 |
| **Household characteristics** |  |  |  |  |  |  |  |  |
| *Sex of household head* |  |  |  |  |  |  |  |  |
| Male | 1.00 |  |  |  |  |  |  |  |
| Female | 0.69 (0.53 - 0.91)* | 0.008 | 0.51 (0.27- 0.99)* | 0.046 | 0.54 (0.25 - 1.18) | 0.122 | 0.69 (0.12 - 3.85) | 0.669 |
| *Quality of housing material* |  |  |  |  |  |  |  |  |
| Good |  |  |  |  |  |  |  |  |
| Average | 1.58 (1.30 - 1.92)* | <0.001 | 0.53 (0.29-0.98)* | 0.041 | 0.27 (0.12-0.63)* | 0.002 | 2.13 (0.57-7.98) | 0.264 |
| Poor | 1.28 (0.94 - 1.75) | 0.122 | 0.25 (0.09-0.72)* | 0.009 | 0.12 (0.03-0.48)* | 0.003 | 0.21 (0.02-2.19) | 0.192 |
| *Wealth index* |  |  |  |  |  |  |  |  |
| Poor | 1.23 (0.94 - 1.61) | 0.13 |  |  |  |  |  |  |
| Middle | 1.04 (0.82 - 1.31) | 0.767 |  |  |  |  |  |  |
| Rich | 1.00 |  |  |  |  |  |  |  |
| *Obtained net from campaigns* |  |  |  |  |  |  |  |  |
| No | 1.00 |  |  |  |  |  |  |  |
| Yes, campaign | 1.39 (0.85-2.27) | 0.053 | 1.77 (1.03-3.04)* | 0.038 | 1.21 (0.62 - 2.32) | 0.577 | 7.25 (2.02-26.05)* | 0.002 |
| ANC / Immunization clinic | 4.33 (2.06 - 9.11)* | <0.001 | 5.30 (2.32 - 12.14)* | <0.001 | 4.32 (1.58 - 11.85)* | 0.004 | 7.94 (1.29-48.81)* | 0.025 |
| *Number of months ago net was obtained* |  |  |  |  |  |  |  |  |
| <=2 vs >2 | 0.86 (0.45 - 1.63) | 0.644 |  |  |  |  |  |  |
| *Number of under5 in the household* |  |  |  |  |  |  |  |  |
| 1 | 1.00 |  |  |  |  |  |  |  |
| 2 | 1.11 (0.92 - 1.34) | 0.285 | 1.05 (0.62-1.76) | 0.865 | 1.13 (0.60-2.13) | 0.695 | 1.31 (0.44-3.89) | 0.628 |
| >=3 | 0.77 (0.62 - 0.94)* | 0.012 | 0.68 0.36-1.28) | 0.229 | 0.71 (0.34-1.49) | 0.37 | 0.73 (0.18-3.07) | 0.671 |
| *No. of household member per net* |  |  |  |  |  |  |  |  |
| <=2 | 1.80 (1.48 - 2.19)* | <0.001 | 0.88 (0.58 - 1.33) | 0.548 | 0.97 (0.59 - 1.58) | 0.887 | 0.97 (0.35-2.72) | 0.957 |
| >2 | 1.00 |  | 1 |  | 1 |  |  |  |
| *Ownership of radio/tv in household* |  |  |  |  |  |  |  |  |
| Yes vs No | 1.07 (0.89 - 1.28) | 0.502 |  |  |  |  |  |  |
| **Community (cluster) characteristics** |  |  |  |  |  |  |  |  |
| *Location of residence* |  |  |  |  |  |  |  |  |
| Rural vs Urban | 1.51 (1.09 - 2.08)* | 0.012 | 0.72 (0.27-1.94) | 0.526 | 0.31 (0.08-1.25) | 0.101 | 3.28 (0.59-18.34) | 0.176 |
| *Community poverty* |  |  |  |  |  |  |  |  |
| Low | 1.00 |  |  |  |  |  |  |  |
| High | 0.87 (0.64 - 1.19) | 0.382 |  |  |  |  |  |  |
| *Community illiteracy* |  |  |  |  |  |  |  |  |
| Low | 1.00 |  |  |  |  |  |  |  |
| High | 1.21 (0.89 - 1.65) | 0.227 |  |  |  |  |  |  |
| *Community disadvantage* |  |  |  |  |  |  |  |  |
| Low | 1.00 |  |  |  |  |  |  |  |
| Medium | 2.08 (1.42 - 3.06)* | <0.001 | 5.18 (1.67 - 16.06)* | 0.004 | 11.37 (2.06 - 62.79) | 0.005 | 3.87 (0.58-25.73) | 0.162 |
| High | 3.53 (2.45 - 5.09)* | <0.001 | 10.91 (3.12 - 38.06)* | <0.001 | 51.67 (8.03-332.52)* | <0.001 | 9.61 (0.87-106.57) | 0.065 |

Table 6

| **Individual child/maternal factors** | **Unadjusted OR (95% CI)** | **p value** | **All States** | | **States with Malaria Increase** | | **States with Malaria reduction** | |
| --- | --- | --- | --- | --- | --- | --- | --- | --- |
|  |  |  | **Adjusted OR (95% CI)** | **p value** | **Adjusted OR (95% CI)** | **p value** | **Adjusted OR (95% CI)** | **p value** |
| *Child's age (Months)* |  |  |  |  |  |  |  |  |
| 0 - 11 | 1.00 |  | 1.00 |  | 1.00 |  | 1.00 |  |
| 12 - 23 | 1.08 (0.76 - 1.54) | 0.677 | 0.94 (0.65 - 1.36) | 0.735 | 1.03 (0.55 - 1.94) | 0.916 | 0.87 (0.54 - 1.39) | 0.561 |
| 24 - 59 | 1.92 (1.40 - 2.64)* | <0.001 | 1.78 (1.28 - 2.48)* | 0.001 | 2.37 (1.36 - 4.13)* | 0.002 | 1.47 (0.97 - 2.22) | 0.073 |
| *Child's sex* |  |  |  |  |  |  |  |  |
| Male |  |  |  |  |  |  |  |  |
| Female | 0.86 (0.72 - 1.04) | 0.114 |  |  |  |  |  |  |
| *Relationship to head of household* |  |  |  |  |  |  |  |  |
| Child | 1.00 |  | 1.00 |  | 1.00 |  | 1.00 |  |
| Grandchild | 1.42 (1.02 - 2.00)* | 0.04 | 1.51 (0.94 - 2.43) | 0.088 | 0.87 (0.35 - 2.13) | 0.756 | 2.03 (1.14 - 3.59)* | 0.016 |
| Other relative | 1.43 (0.88 - 2.34) | 0.153 | 1.72 (0.87 - 3.39) | 0.117 | 2.15 (0.88 - 5.28) | 0.094 | 0.87 (0.27 - 2.80) | 0.821 |
| *Anaemia* |  |  |  |  |  |  |  |  |
| Severe | 15.37 (9.01 - 26.20)* | <0.001 |  |  |  |  |  |  |
| Moderate | 4.92 (3.84 - 6.31)* | <0.001 |  |  |  |  |  |  |
| Mild | 1.98 (1.50 - 2.62)* | <0.001 |  |  |  |  |  |  |
| None |  |  |  |  |  |  |  |  |
| *Use of LLIN (Yes vs No)* | 0.94 (0.77 - 1.14) | 0.514 | 0.84 (0.67 - 1.03) | 0.107 | 0.76 (0.53 - 1.09) | 0.136 | 0.86 (0.65 - 1.13) | 0.284 |
| *Fever in past 2 weeks (Yes vs No)* | 1.33 (1.08 - 1.65)* | 0.008 | 1.31 (1.06 - 1.63)* | 0.013 | 1.74 (1.22 - 2.49)* | 0.002 | 1.13 (0.86 - 1.49) | 0.384 |
| *Medical treatment of fever* | 0.69 (0.47 - 1.00) | 0.047 |  |  |  |  |  |  |
| *Stunting (Yes vs No)* | 1.28 (1.06 - 1.55)* | 0.01 | 1.12 (0.90 - 1.38) | 0.303 | 1.26 (0.89 - 1.79) | 0.187 | 1.05 (0.80 - 1.38) | 0.736 |
| *Wasting (Yes vs No)* | 1.17 (0.81 - 1.69) | 0.39 |  |  |  |  |  |  |
| *Age of mother (Years)* |  |  |  |  |  |  |  |  |
| < 25 | 1.00 |  |  |  |  |  |  |  |
| 25-34 | 1.13 (0.88 - 1.45) | 0.347 |  |  |  |  |  |  |
| 35-49 | 1.19 (0.90 - 1.58) | 0.217 |  |  |  |  |  |  |
| *Mother's education* |  |  |  |  |  |  |  |  |
| None | 3.09 (2.33-4.11) | <0.001 | 1.89 (1.32 - 2.70)* | <0.001 | 1.89 (1.03 - 3.45)* | 0.039 | 1.99 (1.26 - 3.13)* | 0.003 |
| Primary | 2.11 (1.49-2.99) | <0.001 | 1.54 (1.06 - 2.22)* | 0.022 | 1.77 (0.94 - 3.32) | 0.078 | 1.46 (0.92 - 2.32) | 0.111 |
| Secondary/higher | 1.00 |  | 1.00 |  | 1.00 |  | 1.00 |  |

REVIEWER 2:

The manuscript presents data on drivers of LLIN in malaria endemic states in Nigeria. The data is sound and the implication of the study is genuine. However, I think the manuscript should be reread and language should be looked into seriously. Also, the conclusion and implication part at the end should be rewritten to communicate aptly the message in the write up. The abstract can also be improved on. I recommend the paper for publication because it presents information that can be impactful in assisting Nigeria and international funders to focus on drivers of malaria prevalence in the country.

Line 29-30: "The 13 study States were stratified into two based on whether they had increased or reduced parasitaemia between 2015 and 2018.". THIS STATEMENT NEED TO RECASTED FOR CLARITY.

Line 38: "were also more likely of LLIN use". RECAST TO "were also more likely to use LLIN".

Line 43: "were more likely of parasitaemia". RECAST "were more likely to have the parasite".

Line 47-48: "The key drivers of LLIN utilisation mainly related to net source and socioeconomic characteristics which is a key factor for parasitaemia". ALL THE CONCLUSION SHOUKD BE REWRITTEN.

Line 60: World Malaria Report of 2021 should be used instead.

Line 69: The most predominant in Nigeria only or in all regions of the world.

Line 72: I dont think the word "NATIONAL" is needed in the statement "the national prevalence of malaria".

Line 89: Remove the word "THE" in the sentence "in the 13 Nigerian States with"

Line 103: The spelling for "FOCUSED" isnt correct.

Line 216: CHILDREN'S

Line 381: Which other studies?

Line 381-384: The statement need to be recasted

Line 396-398:  It has been shown scientifically that children in areas of high malaria transmission intensity develop age398 related immunity. ANY REFERENCE FOR THIS

Line 411: Remove "THE" in the statement  "management and the use of antimalarials".

Line 469-475: I think this statement should be removed because this isnt part of conclusion.

Line 489: I think the authors should rewrite the whole statement under "IMPLICATIONS OF FINDINGS FOR POLICY AND PROGRAMMES" as the statements looks more of conclusion.
