# Supplementary material for: Drivers of long-lasting insecticide-treated net utilisation and parasitaemia among under-five children in 13 States with high malaria burden in Nigeria
Source: PLoS One. 2022 May 6;17(5):e0268185. doi: 10.1371/journal.pone.0268185 (PMC9075637; doi:10.1371/journal.pone.0268185)
Supplement: Attachment — Submitted filename: R2 PLOS ONE ReviewersComments AND RESPONSES.docx [file pone.0268185.s004.docx]

COMMENTS TO AUTHOR(S)
The manuscript is sound but there is need for minor revision in some components as elaborated in the comments by the reviewers.

REVIEWER 1:
COMMENTS
The author has effected all the corrections pointed out.
Aside that, I noted the following minor corrections which I did not point out the first time (Please I apologize for that).
1. Add title to the first column in table 3. I suggest ‘Variables / categories’
2. Redo the first and second rows of Table 5 as shown below.
3. Redo the first and second rows of Table 6 as shown below
The author can actually lift the Tables (Tables 5 and 6) as presented here. I have only adjusted the rows for the author. I did not touch the data.
The manuscript is recommended for publication.
Thanks.
Dr. Chukwudi Michael Egbuche

RESPONSE: THANK YOU FOR THE SUGGESTIONS. ALL HAS BEEN IMPLEMENTED

REVIEWER 2:
The manuscript presents data on drivers of LLIN in malaria endemic states in Nigeria. The data is sound and the implication of the study is genuine. However, I think the manuscript should be reread and language should be looked into seriously. Also, the conclusion and implication part at the end should be rewritten to communicate aptly the message in the write up. The abstract can also be improved on. I recommend the paper for publication because it presents information that can be impactful in assisting Nigeria and international funders to focus on drivers of malaria prevalence in the country.

THANK YOU FOR THE CONSTRUCTIVE SUGGESTIONS

Line 29-30: "The 13 study States were stratified into two based on whether they had increased or reduced parasitaemia between 2015 and 2018.". THIS STATEMENT NEED TO RECASTED FOR CLARITY.

RESPONSE: IT HAS BEEN RECASTED

Line 38: "were also more likely of LLIN use". RECAST TO "were also more likely to use LLIN".
RESPONSE: DONE

Line 43: "were more likely of parasitaemia". RECAST "were more likely to have the parasite".
RESPONSE: REVISED AS SUGGESTED

Line 47-48: "The key drivers of LLIN utilisation mainly related to net source and socioeconomic characteristics which is a key factor for parasitaemia". ALL THE CONCLUSION SHOUKD BE REWRITTEN.
RESPONSE: REVISED AS SUGGESTED

Line 60: World Malaria Report of 2021 should be used instead.
THANK YOU. THIS WAS NOT YET OUT AS AT SUBMISSION. WE HAVE USED THE 2021 REPORT NOW

Line 69: The most predominant in Nigeria only or in all regions of the world.
RESPONSE: WE HAVE INDICATED NIGERIA

Line 72: I dont think the word "NATIONAL" is needed in the statement "the national prevalence of malaria".
RESPONSE: REVISED AS SUGGESTED. THANK YOU

Line 89: Remove the word "THE" in the sentence "in the 13 Nigerian States with"
RESPONSE: DONE

Line 103: The spelling for "FOCUSED" isnt correct.
RESPONSE: THANK YOU. IT’S BEEN CORRECTED

Line 216: CHILDREN'S
RESPONSE: CORRECTED

Line 381: Which other studies?
RESPONSE: PREVIOUS STUDIES. REFERENCES PROVIDED

Line 381-384: The statement need to be recasted
RESPONSE: REVISED

Line 396-398: It has been shown scientifically that children in areas of high malaria transmission intensity develop age398 related immunity. ANY REFERENCE FOR THIS
RESPONSE: REFERENCE PROVIDED

Line 411: Remove "THE" in the statement "management and the use of antimalarials".
RESPONSE: THANK YOU. REVISED AS RECOMMENDED

Line 469-475: I think this statement should be removed because this isnt part of conclusion.
RESPONSE: STATEMENT HAS BEEN MOVED TO DISCUSSION SECTION. THANK YOU

Line 489: I think the authors should rewrite the whole statement under "IMPLICATIONS OF FINDINGS FOR POLICY AND PROGRAMMES" as the statements looks more of conclusion.
RESPONSE: NECESSARY REVISIONS HAS ALSO BEEN DONE IN THIS SECTION. THOUGH, IT WAS NOT A CONCLUSION
